# Robust and language-independent acoustic features in Parkinson's disease

**DOI:** 10.3389/fneur.2023.1198058

**Published:** 2023-06-13

**Authors:** Sabrina Scimeca, Federica Amato, Gabriella Olmo, Francesco Asci, Antonio Suppa, Giovanni Costantini, Giovanni Saggio

**Affiliations:** ^1^Department of Control and Computer Engineering, Polytechnic University of Turin, Turin, Italy; ^2^Department of Human Neuroscience, Sapienza University of Rome, Rome, Italy; ^3^IRCCS Neuromed Institute, Pozzilli, Italy; ^4^Department of Electronic Engineering, University of Rome Tor Vergata, Rome, Italy

**Keywords:** speech analysis, acoustic features, Parkinson's disease, machine learning, statistical analysis

## Abstract

**Introduction:**

The analysis of vocal samples from patients with Parkinson's disease (PDP) can be relevant in supporting early diagnosis and disease monitoring. Intriguingly, speech analysis embeds several complexities influenced by speaker characteristics (e.g., gender and language) and recording conditions (e.g., professional microphones or smartphones, supervised, or non-supervised data collection). Moreover, the set of vocal tasks performed, such as sustained phonation, reading text, or monologue, strongly affects the speech dimension investigated, the feature extracted, and, as a consequence, the performance of the overall algorithm.

**Methods:**

We employed six datasets, including a cohort of 176 Healthy Control (HC) participants and 178 PDP from different nationalities (i.e., Italian, Spanish, Czech), recorded in variable scenarios through various devices (i.e., professional microphones and smartphones), and performing several speech exercises (i.e., vowel phonation, sentence repetition). Aiming to identify the effectiveness of different vocal tasks and the trustworthiness of features independent of external co-factors such as language, gender, and data collection modality, we performed several intra- and inter-corpora statistical analyses. In addition, we compared the performance of different feature selection and classification models to evaluate the most robust and performing pipeline.

**Results:**

According to our results, the combined use of sustained phonation and sentence repetition should be preferred over a single exercise. As for the set of features, the Mel Frequency Cepstral Coefficients demonstrated to be among the most effective parameters in discriminating between HC and PDP, also in the presence of heterogeneous languages and acquisition techniques.

**Conclusion:**

Even though preliminary, the results of this work can be exploited to define a speech protocol that can effectively capture vocal alterations while minimizing the effort required to the patient. Moreover, the statistical analysis identified a set of features minimally dependent on gender, language, and recording modalities. This discloses the feasibility of extensive cross-corpora tests to develop robust and reliable tools for disease monitoring and staging and PDP follow-up.

## 1. Introduction

Parkinson's disease (PD) is a neurodegenerative disorder caused by progressive and irreversible degeneration in the *substantia nigra pars compacta* region of the midbrain. For reasons not yet fully understood, the incidence of this disease has been rapidly increasing over the last two decades, with current incidence rates ranging from 31 to 328 cases per 100,000 inhabitants (1). Despite being idiopathic, PD is known to be prone to several risk factors, among which age and gender are relevant (2) According to recent evidence, PD chiefly occurs at the age of 57/60, with a risk of developing the disease that rises exponentially with ageing. Gender is also an established risk factor, with a male-female ratio of about 3:2 (3).

PDP usually face several motor and non-motor symptoms (4–6), including tremors at rest, bradykinesia, rigidity, dyskynesia (7), gait impairment (8), postural instability, sleep alterations, and voice disorders. Among these, it was estimated that vocal dysfunctions, usually grouped under the definition of dysarthria and dysphonia (9–11), affect ~90% of the PDP population. The incidence of these alterations, as well as the feasibility of rapid, non-invasive, and inexpensive signal collection, have made voice signal analysis a powerful and effective tool in supporting clinical practice (11, 12).

Indeed, voice production is performed through a complex sequence of movements that shape the excitation source to produce the perceived final sound. Any alteration in the control or execution of these movements turns into measurable alterations of the vocal signal, from which information about the patient's health status can be derived (13). In this context, a broad body of literature has been focused on the development of automatic tools for PDP vocal analysis, with promising results in disease staging, monitoring, and follow-up. Among these, the vast majority of the studies demonstrated the effectiveness of vocal-derived information as biomarkers for neurodegeneration, by employing mainly sustained phonation and continuous speech (14).

Sustained phonation is a simple and easily reproducible task that enhances the analysis of the glottal source vibration. Despite the absence of validated guidelines that clearly indicate which vowel has to be preferred, the vast majority of studies on PD vocal analysis employ the sustained phonation of the vowel /a/ (15). In this context, in Naranjo et al. (16) the authors proposed a model based on 44 acoustic features extracted from the sustained vowel phonation of 80 participants. These features were input to a Bayesian classifier, achieving 75.2% accuracy. Similarly, the studies conducted in (17) and (18) employed speech features from the freely available University California Irvine (UCI) dataset (188 PDP and 64 Healthy Controls—HC) (17) in conjunction with a Support Vector Machine (SVM) and a Logistic Regression Stacking Ensemble, respectively. The models yielded 84 and 92.22% classification accuracy.

Despite the excellent performance associated with the sustained phonation task, the lack of involvement of the articulators makes the exercise somehow simplistic, hence not suitable for a more complete analysis of speech production. In fact, some studies pointed out that reading sentences or monologues can help achieving a deeper insight, as they contain additional motion information and require more precise coordination (19). Indeed, a continuous speech analysis can improve the detection of changes in volume, pitch, and timing. In this context, several studies proved the effectiveness of running speech (20), reading text (21), or isolated word repetitions (22) with average classification accuracies between 80 and 95% (19). Nevertheless, while the sustained phonation task is usually considered to be minimally influenced by language, the information achievable from continuous speech embeds several complexities. Indeed, the same algorithm applied to different languages can provide different results. In this context, although many articles deploy PD speech analysis tools employing participants from various nationalities, only a few studies perform a direct language comparison. Among these, in (23) the authors investigated continuous speech samples from five different nationalities (i.e., Czech, English, German, French, and Italian) and identified changes in voice quality, articulation, and speed of speech. Recently, the authors in (24) carried out an analysis of five datasets in five different languages (i.e., Italian, Hebrew, English, Czech, and Spanish), encompassing 241 PDP and 265 HC. The study aimed not only to perform cross-language experiments, but also to verify the feasibility of a model trained on a heterogeneous dataset including participants from different nationalities and recorded in different conditions. This latter experiment yielded 75% classification accuracy provided by an Extreme Gradient Boosting (XGB) classifier. However, the performance is reported on a 10-fold cross-validation (CV), while no additional tests were performed on previously unseen samples.

In the present work, we aim at identifying the most adequate speech tasks as well as to investigate the most effective and trustworthy features that prove to be robust against external co-factors such as language, gender, and data collection modality. More in detail, we conducted a performance comparison between different types of speech tasks or their combination, to identify a speech protocol that can capture the vocal alterations of parkinsonian patients, while minimizing the effort required to the participants. Moreover, we employed heterogeneous corpora including participants from three different nationalities, and conducted an in-depth statistical analysis to assess the influence of participants and data-collection characteristics on the feature extracted and the model performance.

## 2. Materials and methods

### 2.1. Materials

We employed six different and heterogeneous corpora, including a total of 354 participants (176 PDP and 178 HC) from three different nationalities. For the sake of brevity, in this work we refer to each dataset as the language of the speakers it contains. In the case of multiple datasets under the same nationality, we made a distinction across the city where the corpus was recorded.

The *Italian Bari* corpus refers to the Italian Parkinson's Voice and Speech (IPVS), an open-access dataset that is available under the Creative Commons Attribution License (CC BY 4.0). Inclusion criteria and other information are reported in Dimauro et al. (25).

The *Italian Torino1* and *Italian Torino2* corpora refer to two private datasets recorded by ourselves. Participants were enrolled at *A.O.U Città della Salute e della Scienza di Torino* and *Associazione Amici Parkinsoniani Piemonte Onlus*. The inclusion criteria were: a clinical diagnosis of idiopathic PD with vocal signs and symptoms and no major cognitive impairment or other conditions preventing the patient from correctly accomplishing the task. To evaluate the efficiency of the proposed algorithms in the real-world scenario, both corpora were registered under suboptimal conditions. More in detail, *Italian Torino1* includes samples collected in a non-supervised manner through a web application that guided the users in the execution of the same tasks encompassed in *Italian Bari*. As for *Italian Torino2*, audio samples were collected in a quiet room through an iPhone 12 under the supervision of an operator. Participants were instructed to sit in a relaxed position with the back and arms adhering to the back- and arm-rest (26) and perform the sustained phonation of the vowel /a/ at a comfortable volume with the smartphone placed ~10 cm to the mouth. The data collection of the two datasets were conducted in accordance with the Declaration of Helsinki and approved by the Ethics Committee of the A.O.U Città della Salute e della Scienza di Torino (approval number 00384/2020). Participants received detailed information on the study purposes and execution. Informed consent for the observational study was obtained; demographic and clinical data were noted anonymously.

The *Spanish* corpus refers to the PC-GITA dataset, a private set of vocal recordings whose details are reported in Orozco-Arroyave et al. (27).

The *Czech* corpus is a publicly dataset that is available under the Creative Commons Attribution License (CC BY 4.0). Additional and more detailed information can be found in Hlavnicka et al. (28).

The *Italian Rome* is a private dataset detailed in Suppa et al. (29).

Table 1 summarizes the characteristics of the datasets, including numerosity, composition, and equipment employed during the data-collection procedure.

**Table 1:** Demographic characteristics in the analyzed population.

	**Italian_Bari**	**Italian_Torino1**	**Spanish**	**Czech**	**Italian_Torino2**	**Italian_Rome**
N PDP	26	17	50	22	15	46
N HC	21	13	50	22	15	56
N Male PDP	17	12	25	10	11	33
N Male HC	9	8	25	11	11	15
Recording set-up	Microphone	Smartphone	Microphone	Microphone	Smartphone	Microphone
Age PDP	67.38 ± 6.57	70.35 ± 7.23	61.14 ± 7.78	65.40 ± 9.10	70.38 ± 7, .7	65.00 ± 6.81
Age HC	67.00 ± 4.38	63.62 ± 5.80	60.90 ± 6.80	54.50 ± 17.70	59.93 ± 15.15	50.07 ± 9, 66
Language	Italian	Italian	Spanish (Colombian)	Czech	Italian	Italian
Speech tasks	/a/ + /e/ + Phrases	/a/ + Phrases	/a/ + /e/ + Phrases	/a/	/a/	/e/ + Phrases
*N* Recordings	564	330	800	44	30	408

*N*, Number of subjects.

Data analysis was carried out in Python employing Praat (by Paul Boersma and David Weenink, Phonetic Sciences, University of Amsterdam) for pre-processing and feature extraction. The Parselmouth library was used as an interface to the Praat internal code.

### 2.2. Methods

#### 2.2.1. Pre-processing and feature extraction

Data included in the six corpora were characterized by different sampling rates. Thereafter, all the recordings were firstly down-sampled to 16 kHz to assure consistency. The signal amplitudes were normalized in the range [0, 1] to prevent the speaker-microphone distance from affecting the model. We manually removed the initial and final silence regions; hence, no further preparatory steps were required. Finally, for the analysis of the phrases-repetition task, we employed the Praat software to detect start- and end-points of voiced regions. After identifying and merging voiced regions, we framed each signal into 25 ms windows with 10 ms overlap and extracted features from each segment (30–32). This set encompassed, among the others, *periodicity measures* (i.e., fundamental frequency F0, first three formants, and their bandwidths) and *noise measures* including Harmonc to Noise Ratio (HNR), Cepstral Peak Prominence (CPP), and Glottal to Noise Excitation ratio (GNE). Moreover, we extracted spectral and cepstral features together with their first and second derivative, which often proved to be effective in PD-related speech analysis (15). Finally, we grouped the features into one feature vector and calculated five statistics (i.e., mean value, median value, standard deviation, kurtosis, and skewness). It is worth noting that jitter and shimmer variants were evaluated from the entire signal, given that their definition already embeds a comparison among contiguous frames. Table 2 reports an overview of the employed features together with their description. Min-max normalization was applied to the whole set of features to scale them to the same range.

**Table 2:** Overview of the extracted features.

**Feature**	**Description**
F0	Vocal folds vibration and frequency alteration.
Formants (1–3)	Various indicators related to the estimated formant frequencies, which define the resonances of the vocal tract. They include central frequencies and bandwidths.
Jitter (local, local_absolute, RAP, DDP, PPQ5)	Variation of F0 between cycles.
Shimmer (local, local_dB, APQ3, APQ5, APQ11)	Variation of the peak amplitude between cycles.
Intensity	Intensity contour of the speech signal.
STE	Short-time energy of the speech signal.
**Noise measures**: CPP, HNR, GNE	Extent of noise in speech modeled using linear and non linear-energy concepts as well as the ratio between harmonic and non-harmonic components.
**Spectral features**: flux, skewness, entropy crest, flatness, slope, roll off, spread, centroid, kurtosis	Various features related to the variation in the frequency spectrum.
PLP(1–13) Δ PLP(1–13), ΔΔPLP(1–13)	Perceptual Linear Prediction Coefficients. Coefficients of an all-pole model computed on the inverse transform of aperceptually-scaled, and logarithmically low-passed spectrum.
MFCC(1–13) Δ MFCC(1–13), ΔΔMFCC(1–13)	Mel Frequency Cepstral Coefficients. Obtained by inverse- transforming the Mel scaled of the signal, with each coefficient pertaining to a Mel window. The set of coefficients is usually employed to detect subtle changes in the motion of articulators.
DFA	Measure of the stochastic self-similarity of a signal through de-trending over increasingly numerous windows.

#### 2.2.2. Experiment 1: identification of the speech protocol

For the first aim of this work, i.e., to identify a set of speech tasks that can capture PDP vocal alterations while minimizing the effort required to the patients, we employed the four corpora that included at least two speech exercises (i.e., *Italian Bari, Italian Torino1, Spanish*, and *Italian Roma*). This analysis was carried out in two subsequent steps. First, we assessed whether different variants of the same speech tasks (e.g., different vowels for the sustained vowel phonation, different phrases for the sentence repetition) exhibit different effectiveness in discriminating PDP and HC. If so, we wanted to verify if the same evidence occurs in multiple corpora, regardless of the speakers' language or data-collection modality. Thereafter, we investigated whether different speech tasks or their combination can provide better performance in automatically distinguishing PDP from HC. Due to the composition of the included corpora, we used the *Italian Bari* and *Spanish* datasets to compare /a/ and /e/ vowels. Similarly, we employed *Italian Bari, Italian Torino1, Spanish*, and *Italian Roma* to identify possible difference within a set of phonetically balanced phrases. It is worth noting that the first two datasets included the pronunciation of the same set of sentences, hence, after performing the comparison between the two vowels, we decided to merge them into a single dataset. We remark that, due to the differences in the collection procedure, a stratified fusion was applied to maintain the same proportion of the two datasets in any subsequent split. The best vowels and phrases resulting from this analysis underwent a further comparison to assess whether a single task or their combination can better distinguish between the two classes. The combination of the two tasks is obtained through an early fusion of the related features. Indeed, in a previous work (22) we demonstrated that this approach is more effective than a late fusion through majority voting. Also in this case, the results obtained from different corpora were taken into account to test the robustness of the obtained evidence. The dataset Italian Roma only contains the phonation of a single vowel (/e/), hence this latter was assumed to be the most significant. Due to the balanced distribution of the corpora used in this study, we compared the effectiveness of each speech task as the accuracy obtained from a binary classification model trained with features extracted from the task at hand. The pipeline employed included a feature selection step, performed through the Boruta algorithm, and a classification step.

The Boruta algorithm is a wrapper technique that iteratively uses the classification scores of a Random Forest (RF) model to identify the optimal set of features for the application at hand (33). For each input variable, the algorithm creates a shadow attribute by shuffling the values of the original features across objects. The classification is then performed employing the extended set of features. The chief assumption under the algorithm is that adding randomness to the system and analyzing its impact on the model can highlight the most significant features (34).

As for the automatic binary classification between PDP and HC, we tested ten different algorithms: k-Nearest Neighbors (KNN), SVM, Gaussian Process (GP), Decision Tree (DT), RF, Artificial Neural Network (ANN), Naive Bayes (NB), Linear Discriminant Analysis (LDA), AdaBoost (ADA), and XGB. To minimize the influence of the classification model on the comparison between speech tasks, we decided to average their performance and use the returned value for comparisons. To avoid the weak generalization capability of the model, we performed feature selection and model training on 70% of the original dataset and evaluated the performance through a 10-fold cross-validation (CV). The remaining 30% of participants were employed only during the testing phase, so the speaker independence was guaranteed (i.e., recordings from each participant were either in train or in test), without further optimization.

The speech task yielding the best performance was assessed through the accuracy metric. F1-score, Sensibility, Specificity, and Area Under the Curve (AUC) were also computed for comparison purposes.

#### 2.2.3. Experiment 2: analysis of the features effectiveness and robustness

In the second experiment, we performed a series of statistical tests to evaluate the feature effectiveness in discriminating between PDP and HC and the robustness against external factors. We first applied the *U*-Mann–Whitney test to *Italian Bari, Italian Torino1, Spanish, Italian Torino2*, and *Czech* corpora and identified the features that exhibited a statistically significant difference (*p* < 0.05) between PDP and HC in at least three datasets. Thereafter, we created a unified dataset and repeated the test to study the influence of the dataset heterogeneity on the selected features.

**Figure 1:**
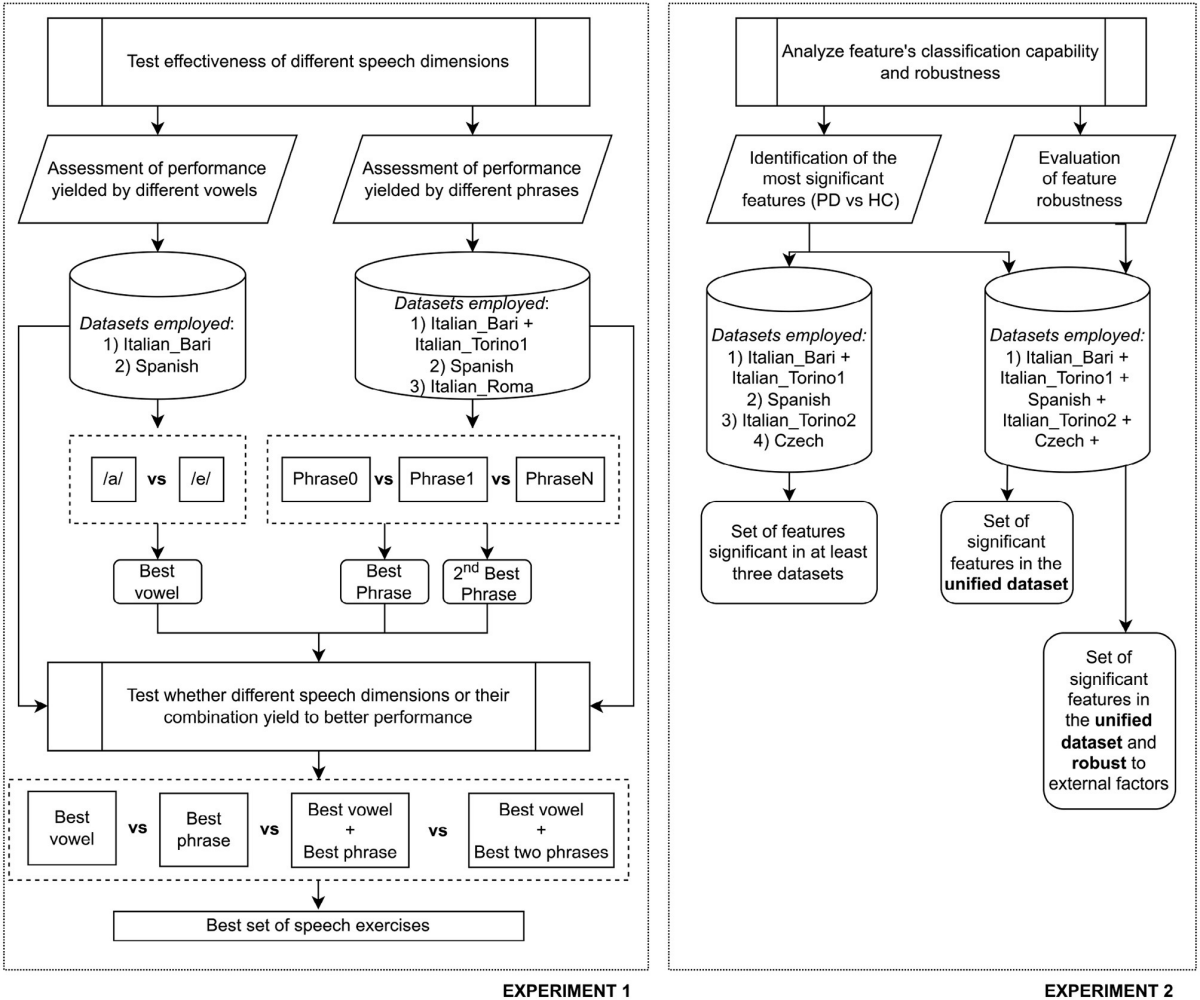
Schematic overview of the two carried out experiments.

Finally, we performed a Kruskal-Wallis test to assess the presence of statistically significant differences (*p* < 0.05) in the feature distribution due to external factors such as gender, language, and the data-collection modality. In order to avoid possible bias due to differences between HC and PDP, we performed the test separately on the two subgroups and considered a feature as exhibiting significant differences only if the null hypothesis was rejected in both populations. Based on the results of the statistical analyses, a resulting set of effective and robust features underwent feature selection and classification steps. The same pipeline employed in Experiment 1 was applied. It is worth noting that only speech samples associated with the sustained vowel phonation were employed for this experiment to minimize possible bias due to language-dependent phonemes. Moreover, also in this case, to avoid the weak generalization capability of the model, we trained the model on 70% of the original dataset and evaluated the performance using a 10-fold CV. The remaining 30% of participants was employed only during the testing phase, without any further optimization. In Figure 1, we report a schematic overview of the two carried out experiments.

## 3. Results

### 3.1. Experiment 1

**Figure 2:**
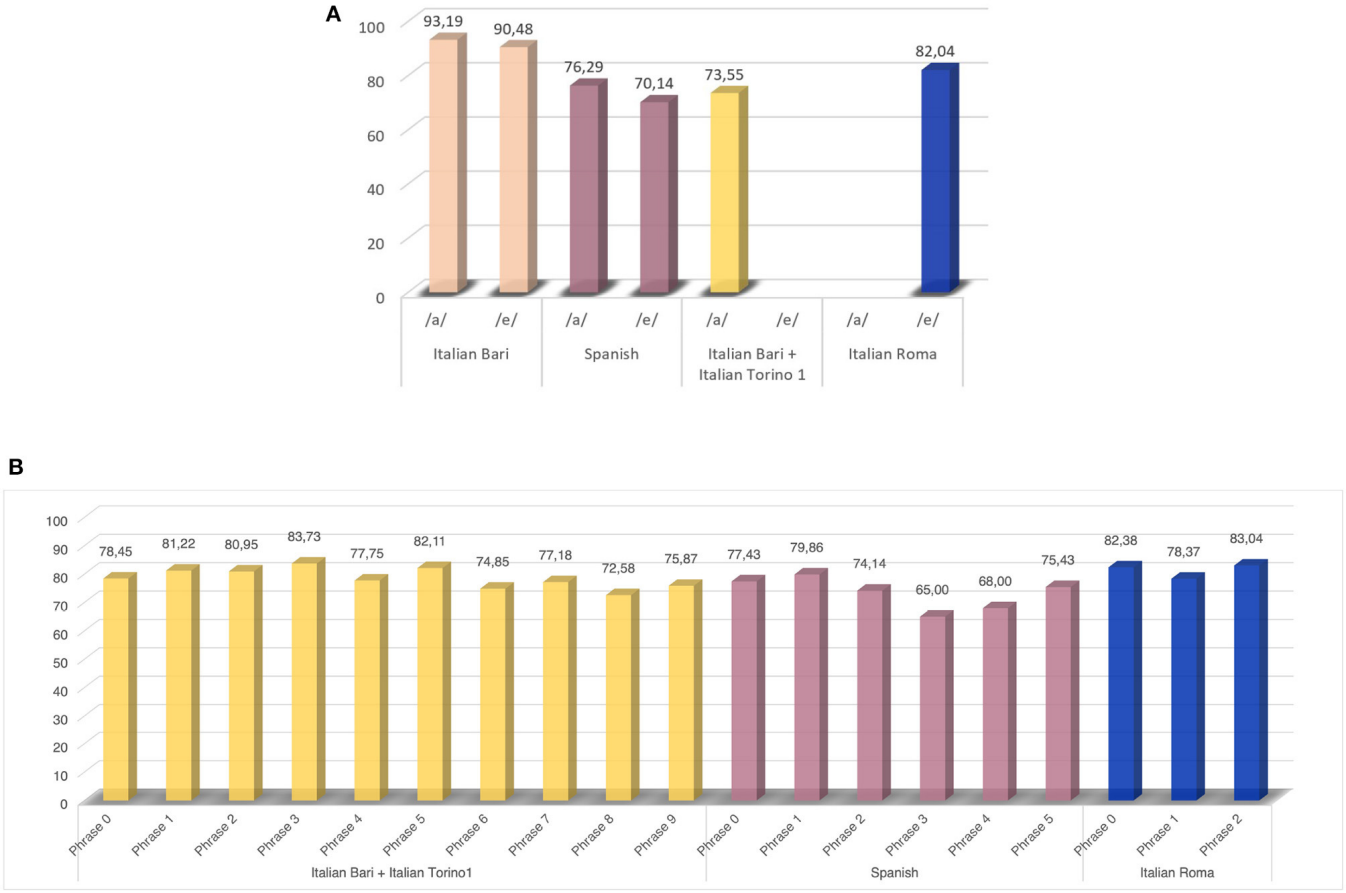
Experiment 1: results of the comparison across different vowels **(A)** and different phrases **(B)** in differentiating between HC and PDP.

**Figure 3:**
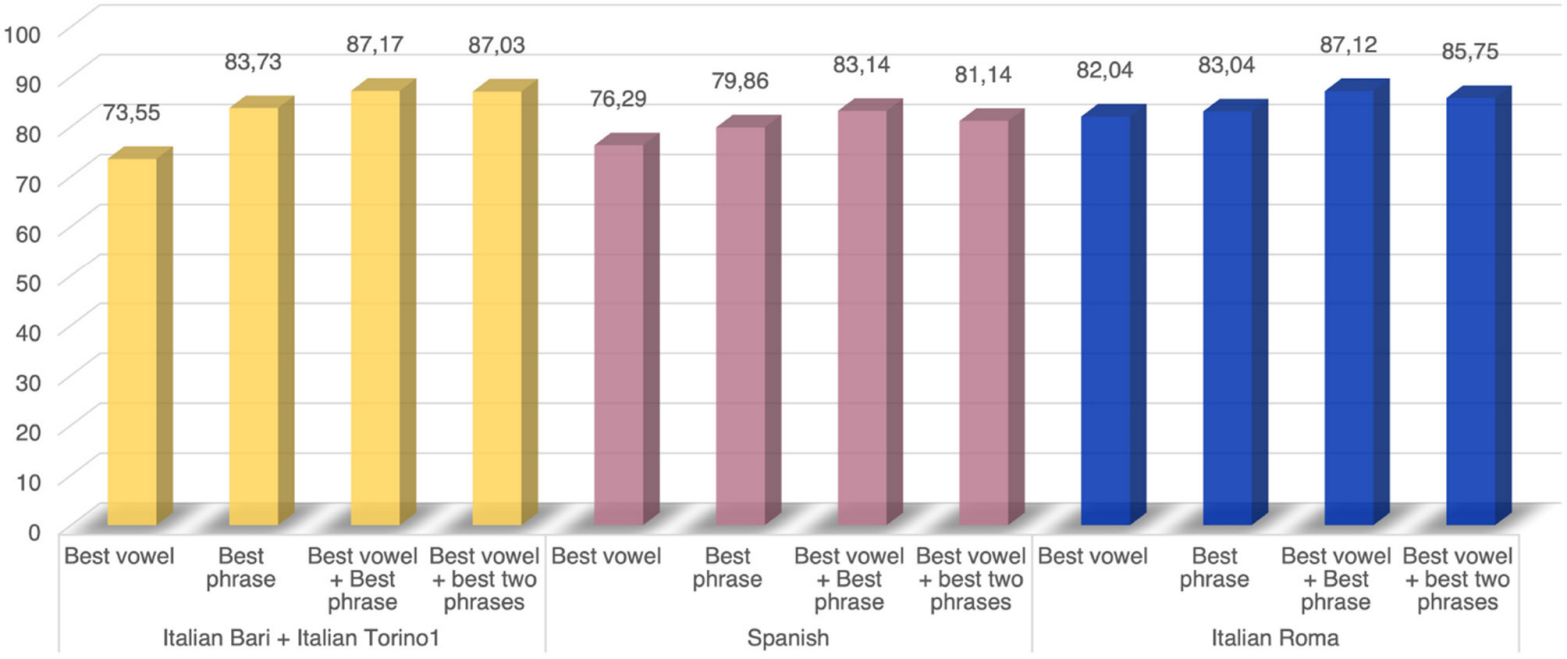
Experiment 1: results of the comparison across different speech tasks or their combination.

In Figure 2, we report the results of the first comparison performed in Experiment 1. In particular, in Figures 2A, B the classification accuracy for vowel and phrase comparisons are depicted. In Figure 3, we report the results of the comparison among the best-performing speech exercises and their combination. After selecting the best speech protocol, we performed an additional comparison in order to identify the classification model that yields the best performance. To this aim, for each model employed, we computed the average classification accuracy across the three tested corpora. According to our analysis, the best models resulted to be KNN and GP, which lead to 91% average accuracy on validation sets. The two best-performing classifiers were then employed to compute a comprehensive set of metrics on validation and tests set, in order to evaluate the capability of the model to predict new and previously unseen samples (Table 3).

**Table 3:** Classification performance for the two best classifiers.

	**Italian Bari + Italian Torino1**						**Spanish**						**Italian Roma**					
	**10-fold CV**	**Test set**					**10-fold CV**	**Test set**					**10-fold CV**	**Test set**				
	**Acc**.	**Acc. test**	**Spec**.	**Sens**.	**AUC**	**F1**	**Acc**.	**Acc test**	**Spec**.	**Sens**.	**AUC**	**F1**	**Acc**.	**Acc. test**	**Spec**.	**Sens**.	**AUC**	**F1**
KNN	91.3	87.5 .	100	78.6	89.3	88	90	73.3	70.6	76.9	73.8	71.4	91.7	80.6	78.6	82.4	80.5	82.4
GP	88.7	87.5	90	85.7	87.9	88.9	90	73.3	64.7	84.6	74.7	73.3	94.5	74.2	71.4	76.5	73.9	76.5
Features selected	**Phrase**: SHIM_APQ5, DFA_COEFF, HNR_mean, SPEC_CENT_GRAVITY_mean, FORM1_mean, MFCC2_mean, ΔΔMFCC5_mean, PLP0_mean, ΔPLP4_mean, HNR_median, SPEC_SKEWNESS_median, PLP6_std, SPEC_KURTOSIS_median, MFCC13_skew, FORM1_median, MFCC2_median **Vowel**: ΔΔMFCC12_kurt, ΔΔMFCC1_skew, ΔΔMFCC6_skew, ΔPLP0_skew, ΔΔPLP0_skew						**Phrase**: GNE_mean, ΔPMFCC5_median, SPEC_CENT_GRAVITY_mean, SPEC_ROLLOFF_std, MFCC3_std, PLP3_std, ΔPPLP3_std, ΔΔPLP1_std, ΔΔPLP3_std, ΔΔPLP6_std **Vowel**: PLP9_mean, PLP6_median						**Phrase**: SPEC_CENT_GRAVITY_mean, FORM3_mean, PLP10_mean, SPEC_DECREASE_median, FORM1_median, FORM3_median, ΔΔMFCC5_median, ΔΔMFCC3_std, PLP2_std, ΔPLP2_std, SPEC_SLOPE_kurt, MFCC4_skew, MFCC7_skew **Vowel**: PLP3_mean, STE_kurt, SPEC_FLUX_kurt, ΔPLP9_skew, SPEC_ROLLOFF_kurt, STE_skew, SPEC_FLUX_skew, SPEC_ROLLOFF_skew					

Results are expressed as percentage values.

### 3.2. Experiment 2

In Table 4, we report the results of the performed statistical tests. For the sake of brevity, in the case of the unified dataset we include only those features that exhibited a statistically significant inter-class difference and proved robust with respect to language, gender, and dataset characteristics. Table 5 reports the classification accuracy of the two best-performing models for two different feature sets. The first one is obtained from the application of the Boruta algorithm to the set of features extracted in the previous analysis (i.e., those selected in at least three corpora or in the unified dataset and minimally influenced by external factors). The second feature set is obtained by adding external co-variables (i.e., gender, language, data collection modality) prior to the feature selection procedure. In fact, it has been previously demonstrated that the addition of such co-factors can improve the generalization capability of the feature selection procedure (15). For each feature sets obtained from the application of the Boruta algorithm, we performed a *post-hoc* analysis through the computation of the Spearman correlation coefficient, in order to investigate their importance in the application at hand. According to our findings, the standard deviation of the *first formant* showed the highest correlation with class (*r*:0.40, *P*: ≤ 0.001), followed by mean value of the *spectral center of gravity* (*r*:−0.35, *P*: ≤ 0.001) and the *local Jitter* (*r*:0.27, *P*: ≤ 0.001).

**Table 4:** Results of the statistical analysis.

**Present statistically significant differences between PD and HC in at least three dataset (*p* < 0.05)**	**Present statistically significant differences between PD and HC in the unified dataset (*****p***<**0.05)**	
	**Robust to language, dataset type and gender (*****p*** > **0.05)**	**Robust to language and dataset type (*****p*** > **0.05)**
JIT_L, HNR_mean, SPEC_CENT_GRAVITY_mean, MFCC2_mean, MFCC3_mean, PLP2_mean, ΔPLP1_mean, MFCC2_median, HNR_std, SPEC_CENT_GRAVITY_std, FORM1_std, FORM_BAND1_std, ΔPLP1_std, ΔΔPLP1_std, ΔΔPLP2_std, STE_kurtosis, SPEC_FLUX_kurt, SPEC_ROLLOFF_kurtosis, HNR_skew, STE_skew, CPP_skew, SPEC_FLUX_skew, SPEC_ROLLOFF_skew, ΔMFCC11_skew	MFCC4_mean, MFCC4_median, ΔΔMFCC7_median, ΔMFCC13_median, ΔΔMFCC13_median, ΔΔMFCC7_skew	JIT_L, JIT_LAb, FORM_BAND1_median, PLP6_median, MFCC5_skew

**Table 5:** Results of the classification step performed on the unified dataset.

		**10-fold CV**	**Test set**					**Features selected**
		**Acc**.	**Acc. test**	**Spec**.	**Sens**	**AUC**	**F1 score**	
Feature set 1	XGB	71.8	64.9	70.2	60	65.13	64	ΔΔMFCC7_median, JIT_L, PLP6_median, SPEC_CENT_GRAVITY_ mean, FORM1_std
Feature set 2	RF	70.2	70.1	75.8	65.9	70.8	71.6	SPEC_CENT_GRAVITY_ mean, SPEC_CENT_GRAVITY_ std, FORM1_std

## 4. Discussion

Our study confirmed the feasibility of an automatic classification between HC and PDP, identified a series of speech exercises effective in capturing vocal alterations in PDP, and tested the robustness of acoustic features against external co-factors such as language, gender, and data-collection modality.

According to our findings, an adequate speech protocol should include the phonation of a sustained vowel and the pronunciation of a phonetically balanced phrase. As shown in Figure 3, this combination yields a performance improvement up to 13.6% compared to the single vowel phonation and up to 5.08% compared to the pronunciation of a single phrase. Due to the high complexity of the articulatory dimension of the speech process, we performed an additional comparison embedding the second best-performing phonetically balanced sentence. However, no performance improvement was obtained in any of the employed datasets. As for the specific type of speech tasks, the vowel /a/ exhibited better discriminatory capability between PDP and HC than the vowel /e/Figure 2A. Despite the same trend can be observed in the *Italian Bari* and the *Spanish* datasets, this latter achieved lower classification performance, probably due to the different dataset composition. Moreover, despite the the fact that the same algorithm was employed for all the datasets included in the experiment, different performance was registered across different corpora. Indeed, from a comparison of Figure 2A and Figure 3, we can observe a non-negligible performance impairment associated with the single vowel phonation when adding the *Italian Torino1* to the *Italian Bari* corpus. More in detail, in Figure 2A we report the performance obtained on the *Italian Bari* dataset both alone and in conjuction with the *Italian Torino 1* corpus. According to our findings, despite a careful stratification procedure was employed, the inclusion of samples from the latter corpus led to an impairment of the classification accuracy. Despite the need of larger datasets to obtain statistically relevant results, this evidence points out the impact of the data collection modality. In fact, as previously demonstrated in (35) and in (36), despite a careful stratified merging procedure, the usage of vocal recordings collected in different conditions impacts the classification performance.

As for the comparison between different phonetically balanced phrases, our results identify several sentences yielding optimal performance (see Figure 2B). However, except for the *Italian Roma* corpus that includes only three sentences, more than 10% improvement can be achieved by moving from the less- to the most-effective sentence. As for the *Italian Bari* and the *Italian Torino1* corpora, the best performance is associated to *phrase 3* (“Patrizia ha pranzato a casa to Fabio”—“Patrizia had dinner at Fabio's house”), while the worst performance is associated to *phrase 8* (“Marco non è partito”—“Marco did not leave”). The comparison between these two sentences put into evidence the need for a more articulated task to effectively capture vocal alterations, as well as the need of embedding both occlusive and fricative sounds, as previously demonstrated in (36) and (37). Similarly, the comparison between *phrase 1* (“Omar, que vive cerca, trajo miel”—“Omar, who lives near, brought honey”) and *phrase 3* (“Los libros nuevos no caben en la mesa de la oficina”—“The new books do not fit in the office's table”) in the *Spanish* datasets, highlights that better performance is achieved using a sentence that includes more complex and articulated sounds, as well occlusive consonants.

The results of the classification performance yielded by the combination of best-performing vowel and sentence are reported in Table 3. KNN and GP proved to be the best classification models, with classification accuracy on a 10-fold CV ranging from 88.7 to 94.5%. No significant performance impairment is observed when moving to the test set, suggesting an optimal generalization capability. Moreover, the model exhibited similar performance on three different corpora, showing a good robustness. As for the selected features, the Boruta algorithm returned different sets of features for the three corpora, with a high prevalence of phrase-derived features. The comparison between the three subgroups suggests the effectiveness of spectral center of gravity, MFCC, and PLP coefficients.

As for the second experiment conducted in this study, in Tables 4, 5 we reported the results of the feature statistical analysis and the performed classification step, respectively. MFCC proved to be effective in discriminating between PDP and HC also in presence of heterogeneous datasets and did not show a statistically significant correlation with language, gender, or dataset taken into consideration. Similarly, despite F0 did not prove robust against external factors, the related differential amplitude measures (represented by Jitter features) is crucial in discriminating between the two classes and little influenced by language and dataset type. As for the classification step, XGB and RF outperformed the other considered classification models. A generalization capability improvement is associated to the use of feature set 2, which includes external co-variable such as language and gender before feature selection. Indeed, as previously reported (15), we assume that adding such co-factors before feature selection can provide pivotal clues during the procedure, thus leading to a final set of features that are more representative of the actual vocal alterations despite the peculiarities of the population considered. Also in this case, no significant performance impairment is observed when moving from the validation to the test set.

As expected, a performance impairment can be observed in Experiment 2 with respect to Experiment 1 (average classification accuracy on the validation sets of 77.3 and 70.2% in the two Experiments). Despite this impairment, which can be mainly ascribable to the heterogeneity of the dataset, the result confirms the feasibility of a classification algorithm trained on a unified dataset. Indeed, it is well known that one of the main problems encountered in the design of automatic tools for vocal pathology assessment concerns the database size, which can lead to feature selection and classification results excessively fitted on the population at hand (38). Moreover, although the use of highly homogeneous corpora would achieve better results, the same conditions can be hardly repeatable, thus limiting the actual usefulness in real-world scenarios. In this context, the statistical tests performed in Experiment 2, despite not exhaustive, provide pivotal clues on the influence of external co-factors on the acoustic features. This can help identifying those aspects against which stratification is necessary, and those aspects that may be managed deploying a proper algorithm (e.g., through a co-variable introduction before feature selection).

Despite the lack of similar studies in the related literature, we compared our classification results with those in (24). To the best of our knowledge, this is the only similar study conducted up to date. In this work, the authors achieved 75% classification accuracy on a 10-fold CV performed on a heterogeneous dataset including 241 PDP and 265 HC. However, additional analyses on a separate test set were not performed.

## 5. Limitations

Despite the promising results, we acknowledge the presence of several limitations that still must be addressed. Among these, we must mention the numerosity of the included corpora. In fact, despite the cardinality of the datasets used for the analysis allows to achieve statistically significant results and is generally higher than in similar studies, we plan to further increase the sample and test our findings on wider corpora. Moreover, due to the absence of a single protocol performed by speakers of different nationalities and collected in different conditions, we had to perform the second Experiment only on recording from sustained phonations, despite the evidence deriving from Experiment 1 suggests that the contribution of the repetition of a phonetically balanced phrase is crucial. Finally, in both Experiments, we performed binary comparisons and classification, while not addressing any disease staging task.

## 6. Conclusions and future works

In this work, we presented a comprehensive analysis to identify an adequate set of speech exercises that can effectively describe vocal alterations in patients with PD. The results on achieved on different corpora, including participants from different nationalities, showed that the joint use of the sustained phonation and the repetition of a phonetically balanced phrase should be preferred to the use of a single task. As for the specific tasks, the usage of the vowel /a/ together with an articulated sentence that embeds both occlusive and fricative sounds resulted effective, with 91% average classification accuracy on the three datasets. A *post-hoc* analysis of the features selected and input into the ML models revealed different sets for the three corpora, with a high prevalence of phrase-derived features. The comparison between the three subgroups indicated the effectiveness of spectral center of gravity, MFCC, and PLP coefficients.

The second aim of this study was to investigate the feasibility of a classification between HC and PDP employing a heterogeneous dataset and derive information about the feature trustworthiness and robustness against the speaker characteristics and the data-collection modalities. To this aim, we employed 4 corpora including participants from three different nationalities. According to our evidence, MFCC coefficients proved to be effective in discriminating between PDP and HC and did not show a statistically significant correlation with gender, language, and dataset type taken into consideration. Similarly, differential frequency measures represented by Jitter features proved to be crucial in discriminating between the two classes while being influenced only by gender of the speaker. As for the classification step, including external co-variables (such as language and gender before feature selection) improved the generalization capability of the model, yielding 70% accuracy both on train and test sets. Despite a 7% reduction with respect to the single datasets, these results disclose the option for a model trained on unified corpora. Indeed, one of the main limitations of the automatic tools for supporting clinical practice concerns data availability. The evidence from this work can be used to derive information about the external factors that have a stronger influence on the features extracted and the model performance, thus performing a specific stratification and improving the performance of the overall algorithm. In this context, future works will address the collection of additional datasets to increase the overall cardinality and test whether building gender- or data-collection-specific models can improve the classification performance. Moreover, we only performed the multi-lingual analysis on the vowel /a/ in order to control the sources of system complexity (i.e., difference encountered in diverse language may be due to the phoneme-specific characteristics), however, future studies will include also text-based analysis which, according to the results obtained on the single datasets, we expect to provide a performance improvement. Indeed, as previously reported in (39), although a broad body of literature pointed out the effectiveness of an analysis based on phonatory aspects of speech production, more recent studies highlighted the importance of including articulatory aspects as well, in order to allow a better understanding of the impairment. In fact, if on one hand the more explicit hallmarks of PD vocal alterations can be detected through a simple sustained vowel phonation, more complex tasks can reveal hidden clues of the vocal dimensions, such as the difficulty in performing finer movements than can be measured only in presence of a voiced/unvoiced traits alternations (36). At the same time, more complex tasks instrinsically embed several complexities that still have to be addressed, such as the difference in phonemes pronunciation which characterize different languages. In this context, future work will be in the direction of defining a speech protocol that, while including relevant phonetically balanced sounds which can inspect PDP vocal alterations, can be performed also by participants from different nationalities and cultural backgrounds. Finally, in both Experiments, we performed binary comparisons and classifications. Aware of the importance of the disease staging task, future work will also introduce information about the patient's health status and generalize the current evidence in the presence of multi-class analyses.

## Data availability statement

Publicly available datasets were analyzed in this study. This data can be found here: the Italian Bari dataset used in this study can be found at: https://ieee-dataport.org/open-access/italian-parkinsons-voice-and-speech; the Czech dataset used in this study can be found at: https://figshare.com/articles/dataset/Synthetic_vowels_of_speakers_with_Parkinson_s_disease_and_Parkinsonism/7628819?file=14172821 vowels of speakers with Parkinson's disease and Parkinsonism/7628819.

## Ethics statement

The studies involving human participants were reviewed and approved by Ethics Committee of the A.O.U Città della Salute e della Scienza di Torino (approval number 00384/2020). The patients/participants provided their written informed consent to participate in this study.

## Author contributions

FAm and GO: conceptualization. SS and FAm: methodology, writing-original draft, software, formal analysis, investigation, writing-review, and editing. SS: resources. GO, AS, GS, GC, and FAs: data curation. FAm, GO, GC, and GS: supervision and project administration. All authors have read and agreed to the published version of the manuscript.
